# Pancréatite aiguë médicamenteuse: à propos de 10 cas

**DOI:** 10.11604/pamj.2017.28.80.12160

**Published:** 2017-09-27

**Authors:** Houcine Maghrebi, Rami Rhaeim, Anis Haddad, Amin Makni, Jouini Mohamed, Kacem Montasser, Ben Safta Zoubeir

**Affiliations:** 1 Faculté de Médecine de Tunis, Université Tunis El Manar, Service de Chirurgie Générale A, Hôpital La Rabta, Tunisie

**Keywords:** Pancréatite, médicamenteuse, pharmacovigilance, Pancreatitis, drug, pharmacovigilance

## Abstract

La pancréatite aiguë médicamenteuse représente environ 2% des pancréatites aiguës. Son incidence est actuellement en augmentation avec plus de 260 médicaments incriminés. Cependant, cette pathologie reste encore peu rapportée dans la littérature, car se pose le problème de l'imputabilité. Nous rapportons notre expérience à travers une série de 10 patients colligés sur une période de 7 ans. La présentation clinique de la PA était souvent non univoque. Le score de Ranson variait de 0 à 5. Nous avons recensé 5 cas de pancréatites œdémateuses et 5 cas de pancréatites nécrotico-hémorragiques. Ces pancréatites étaient souvent résolutives et sans récidive après arrêt définitif du médicament incriminé.

## Abstract (en)

Drug-induced acute pancreatitis (AP) accounts for approximately 2% of acute pancreatitis. Its incidence is increasing, with more than 260 incriminated drugs. However, very few cases have been described in the literature due to accountability problem. We report our experience with 10 cases whose data were collected over a period of 7 years. Clinical presentation of AP was often equivocal. Ranson’s score ranged from 0 to 5. We recorded 5 cases of edematous pancreatitis and 5 cases of necro-bleeding pancreatitis. These pancreatitis were often successfully treated without recurrence after discontinuation of the incriminated drug.

## Introduction

La pancréatite aigüe (PA) est une affection grave qui peut mettre en jeu le pronostic vital. En Tunisie l'étiologie lithiasique est la plus fréquente. La pancréatite aiguë médicamenteuse représente environ 2% des pancréatites aiguës. Son incidence est actuellement en augmentation avec plus de 260 médicaments incriminés [1, 2]. L'évolution est souvent, bénigne mais de rares cas de décès ont été rapportés. Le but de ce travail est de rappeler les caractéristiques épidémiologiques, cliniques, radiologiques et de prise en charge des pancréatites aigues médicamenteuses à travers l'étude de 10 observations.

## Méthodes

Il s'agit d'une étude rétrospective qui a colligé tous les patients présentant une pancréatite aigue d'origine médicamenteuse sur une période de 7 ans, au service de chirurgie générale A de l'hôpital La Rabta.

## Résultats

Nous avons colligé 10 malades hospitalisés pour pancréatite aigüe d'origine médicamenteuse. La présentation clinique de la PA était souvent non univoque. Le taux des lipases dans le sang était toujours supérieur à 3xN. Le délai entre la survenue de la pancréatite et la prise médicamenteuse variait de 0 à 9 jours. L'implication du médicament dans le déclenchement d'une pancréatite s'est basé sur un faisceau d'arguments anamnestique, clinico-biologique et morphologique, en particulier la chronologie exacte de l'évolution des symptômes par rapport à l'administration. Pour chaque malade 2 échographies abdominales avaient infirmé la présence de lithiase vésiculaire. Le bilan phosphocalcique et lipidique était correct pour tous les malades. Le score de Ranson variait de 0 à 5. Nous avons recensé 5 cas de pancréatites œdémateuses et 5 cas de pancréatites nécrotico-hémorragiques Les médicaments incriminés étaient: l'immurel pour 2 malades, le chlorothiazide, l'allopurinol, le Glivec pour les PA oedémateuses et le Lannate, le valproate de sodium, le furosémide, le méthyldopa et l'association triaméterene-méthylchlorothiazide pour les pancréatites aigues nécrotico-hémorragiques (Tableau 1). Aucun malade n'a présenté une suppuration des coulées de nécrose pancréatique. L'évolution était favorable pour tous les malades.

**Tableau 1:** Liste des médicaments incriminés de notre série

Cas	Agents incriminés
1	Immurel
2	Chlorothiazide
3	Allopurinol
4	Immurel
5	Glivec
6	Lannate
7	Valproate de Na
8	Furosemide
9	MéthyDopa
10	Chlorothiazide

## Discussion

La pancréatite aigüe médicamenteuse est une affection rare et qui peut être grave. Elle est définie par la survenue d'une poussée de pancréatite survenant peu après l'introduction d'un médicament ou après augmentation de ses doses, et ce en l'absence d'une cause classique de pancréatite (par ordre de fréquence sous nos cieux: lithiase biliaire, métabolique et alcoolique) [3]. Cette pathologie incite à une recherche de pharmacovigilance avancée. La pancréatite médicamenteuse représente 1-2% de toutes les pancréatites [1, 4]. Il n'existe pas de données actuelles sur la prévalence en Tunisie. Elles sont le plus souvent bénignes et d'évolution favorable comme le cas de nos malades. Beaucoup de produits sont imputés à la survenue de pancréatite médicamenteuse: allergiques, toxiques, idiosyncrasiques. Il existe actuellement 261 médicaments connus pancréatotoxiques. Le mécanisme physiopathologique n'est pas clairement élucidé. Il pourrait s'agir d'une réaction immunoallergique, une cytotoxicité directe sur les cellules pancréatiques ou d'une ischémie de la glande pancréatique [2]. Les antimitotiques restent les médicaments les plus pourvoyeurs de ce genre d'affection. La PA reste néanmoins un diagnostic d'élimination. L'implication d'un médicament dans le déclenchement d'une pancréatite se base sur un faisceau d'arguments. La chronologie exacte de l'évolution des symptômes par rapport à l'administration, l'arrêt et éventuellement la réintroduction du médicament reste l'élément essentiel pour l'orientation diagnostique [5]. En effet, aucun critère sémiologique n'est spécifique d'une PA médicamenteuse. Aucun dosage biologique n'est évocateur du diagnostic. Plus le délai d'apparition de la PA est court par rapport à la prise médicamenteuse, plus l'imputabilité est probable. Et plus les symptômes disparaissent à son arrêt et reprennent à son introduction, plus le produit est incriminé. Mallory et al [6] avaient établi un score d'imputabilité afin d'aider à classer le degré d'imputabilité en possible, probable, ou certaine. Trivedi et al [7] avaient proposé trois classes' Ces pancréatites souvent œdémateuses sont résolutives et sans récidive après arrêt définitif du médicament incriminé. L'évolution est généralement favorable comme l'illustre le cas de nos patients.

## Conclusion

La pancréatite médicamenteuse est une entité actuellement bien reconnue et représente 2% des cas de pancréatite aiguë. Le diagnostic de pancréatite médicamenteuse reste cependant un diagnostic différentiel sous nos cieux là où il faut toujours éliminer d'abord l'étiologie lithiasique. Il est cependant du devoir de chaque professionnel de la santé de déclarer à un centre de pharmacovigilance toute suspicion de pancréatite médicamenteuse.

### Etat des connaissances actuelle sur le sujet

- La pancréatite aigüe médicamenteuse est une affection rare et qui peut être grave;
- Ces pancréatites sont résolutives et sans récidive après arrêt définitif du médicament incriminé.

### Contribution de notre étude à la connaissance

- La pancréatite médicamenteuse représente 1-2% de toutes les pancréatites;
- Son incidence est actuellement en augmentation avec plus de 260 médicaments incriminés;
- L'évolution est généralement favorable comme l'illustre le cas de nos patients.

## Conflits d’intérêts

Les auteurs ne déclarent aucun conflit d'intérêts.

## References

[1] Mc Donald KB, Garber B, Perreault M (2002). Pancreatitis associated with simvastatin plus fenofibrate. Ann Pharmacother..

[2] Tysk C, Al-Eryani AY, Shawabkeh AA (2002). Acute pancreatitis induced by fluvastatintherapy. J Clin Gastroenterol..

[3] Rebours V (2014). Acute pancreatitis: an overview of the management. Rev Med Interne..

[4] Tenner S (2010). Drug-induced acute pancreatitis: underdiagnosis and overdiagnosis. Dig Dis Sci..

[5] Biour M, Delcenserie R, Grange JD, Weissenburger J (2001). Pancréatotoxicité des médicaments. Gastroenterol Clin Biol..

[6] Mallory A, Kern F (1980). Drug-induced pancreatitis: a critical review. Gastroenterology..

[7] Trivedi CD, Pitchumoni CS (2005). Drug-inducedpancreatitis: an update. J Clin Gastroenterol..

